# Challenges in flap monitoring with indocyanine green angiography in experimental models

**DOI:** 10.1016/j.heliyon.2024.e36034

**Published:** 2024-08-14

**Authors:** Mert Ersan, Burak Kaya, Arda Özdemir, Aygül Durdurur Çin, Hakan Ergün

**Affiliations:** a Ankara University Faculty of Medicine, Plastic Reconstructive and Aesthetic Surgery Department, 06110, Turkiye; b Ankara University Faculty of Medicine, Department of Medical Pharmacology, 06110, Turkiye

**Keywords:** Aluminum, Dorsal flap, Flap perfusion, Indocyanine angiography, McFarlane flap, SPY

## Abstract

**Introduction:**

Non-invasive angiography with indocyanine green dye facilitates the assessment of flaps. Although ICG angiography has been successfully utilized in clinical settings for human beings, its application in experimental models exhibits certain limitations. This study aimed to address the encountered issues in angiography with different experimental models and introduce a novel modification to the ICG imaging of the McFarlane flap.

**Materials-methods:**

Rats were randomly divided into three groups: the first group received an epigastric flap (n = 4), the second group received a deep inferior epigastric perforator flap (n = 4), and the third group received a dorsal flap (n = 8). In the first group, sterile silicone background was placed under two flaps. In the second group, no background was used. In the third group, a sterile silicone or aluminum foil was placed under the flaps. Flap perfusions were assessed using fluorescent imaging after flap adaptations, at postoperative 30th minute and 3., 5. and 7. days. Necrosis calculations were performed using all images obtained from the digital camera and the fluorescent imaging. In the third group, the flow velocities were also calculated. All flaps were sent for histopathological examination.

**Results:**

Even with a silicone background, clear perfusion evaluation and determining the threshold value for predicting necrosis rates were challenging. Moreover, a portion of the flaps without background material survived as grafts. Using an aluminum foil background improved image quality by reducing reflection from interior organs.

**Conclusion:**

The use of an aluminum foil background is beneficial in non-invasive angiography for assessing flap perfusions accurately and predicting necrosis in experimental animal models.

## 1 Introduction

Flap surgery is one of the most fundamental methods used in the plastic and reconstructive surgery. Experimental models are used to understand the physiology of flap surgery, to evaluate ischemia, and to investigate treatment options for ischemia related complications [1]. Oksar et al. described the perforator-based flap model, Petry et al. explained the anatomy of the epigastric flap model and in 1965, McFarlane described the dorsal flap model in which the flap was elevated from the level of the deep muscle fascia and supplied by a random pattern in rats [[2], [3], [4]]. Since then, many modifications of this dorsal flap model have been performed and these flaps have been used in many studies related to flap physiology, flap viability and revascularization [1]. The main aim in these studies is to find out the pathophysiology of the flap necrosis and test certain physical or pharmacological attempts for overcoming the complications, like necrosis. It is important to detect or predict these complications as early as possible, with appropriate methods. The introduction of indocyanine green (ICG) angiography to evaluate the viability of flaps since the early 2000s has made ICG angiography a focal point in flap surgery [5]. Although ICG angiography has been successfully used clinical settings, its application in experimental models presents certain limitations. According to our experience, in small experimental animals, particularly those used in skin flap surgery studies, the limited thickness of the dermal layer from which the flap is harvested can result in reflections from underlying tissues. These reflections impede the accurate assessment of flap viability using ICG angiography in such models. Consequently, these limitations in flap models can restrict experimental applications and research, potentially hindering the progression to clinical practice.

In this study, we delve into the application of ICG angiography across three distinct experimental models, emphasizing encountered challenges. Additionally, we introduce the innovative use of a sterile aluminum background in the McFarlane flap model as a strategic solution to address these challenges. This research aims to contribute to the refinement of flap surgery techniques and enhance our ability to predict and manage complications through a meticulous exploration of ICG angiography in diverse flap models.

## 2 Materials - methods

This study was conducted following the ethics committee approval granted by Ankara University Animal Experiments Local Ethics Committee. 16 male Wistar-Albino rats weighing 230–280 g were used. The rats were randomly divided into three groups. An epigastric flap to the first group (n = 4), a deep inferior epigastric perforator (DIEP) flap to the second group (n = 4) and a dorsal flap to the third group (n = 8) were applied. General anesthesia using ketamine (Ketalar®, Pfizer, Turkey, 90 mg/kg) and xylazine (Xylazinbio® 2 %, Bioveta, Turkey, 10 mg/kg) was applied. A jugular catheter was placed to administer ICG dye intravenously to all rats before the flap operation.

In the first group, an axial pattern flap measuring 5 × 3 cm, including a distal area of 1 × 1 cm presumed to have random blood supply, was raised over the right superficial epigastric vessels of rats. Two rats in this group had a sterile silicone background (Esmarch®, Corvus, Turkey) measuring 5 × 3 cm placed under the flaps. In the second group, a 6 × 4 cm DIEP flap was elevated while preserving all existing perforators on the abdominal wall. Subsequently, all perforators except the right second caudal perforator were sacrificed, and the flap was raised based on a single perforator without the use of a background. (Fig. S1).

**Fig. 1:**
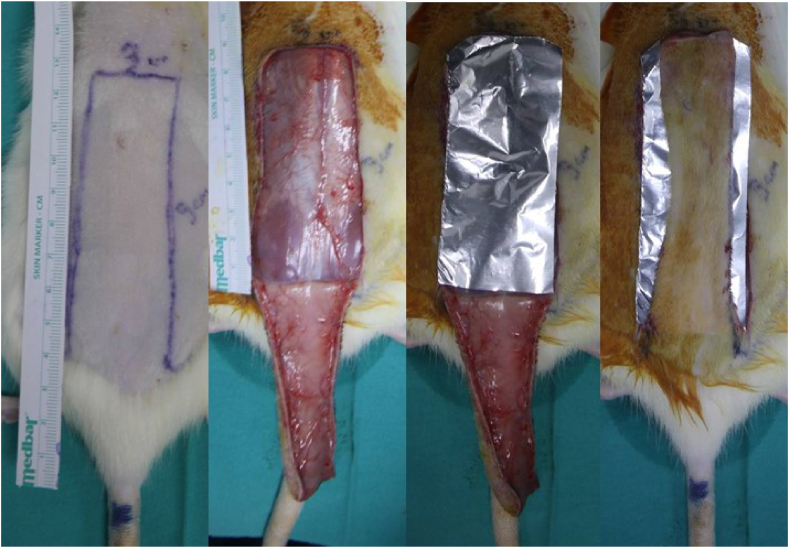
The picture illustrates the stepwise preparation of a random pattern dorsal flap with an aluminum foil background. The preoperative marking of the flap in the size of 9 × 3 cm, the elevation of the random pattern dorsal flap above the deep muscle fascia, the placement of a sterile 9 × 3 cm aluminum foil under the flap, and finally the adaptation of the flap is shown respectively.

In the third group, dorsal flaps of 9 × 3 cm in size, based on a caudal-based random pattern, were elevated. For this group, either a sterile silicone background (applied to four rats) or a sterile aluminum foil background (applied to four rats) measuring 9 × 3 cm was placed under the flap. The dorsal flap model with a sterile aluminum foil background, sized 9 × 3 cm, is illustrated in Fig. 1. All flaps in each group were sutured back by using 3/0 silk sutures (Dogsan, Turkey), spaced 1 cm apart.

ICG dye was administered to each rat at a concentration of 0.25 mg/mL in a volume of 0.1 mL for each imaging session. After the flaps were sutured back, the flow of the flaps was recorded using the SPY device (Novadaq Technologies Inc., Canada) following intravenous administration of ICG (Aurogreen™, Aurolab, India). Threshold values (contour levels) were determined from these images, and the device-recorded estimated necrosis rates were recorded. Imaging was repeated at 30 min after flap adaptation and on postoperative days 3, 5, and 7. In our study, a careful approach was taken towards rat anesthesia, prioritizing ethical treatment and the well-being of the animals. Examining flaps at specific intervals, including 30 min post-surgery and on days 3, 5, and 7, was a deliberate choice, enabling prompt and strategic observations of flap perfusion and viability. The decision to begin assessments at 30 min aimed to capture initial responses, facilitating a nuanced comprehension of flap behavior. Focusing on days 3, 5, and 7 for indocyanine green (ICG) imaging is consistent with findings from our preliminary experiments, which improves assessment efficacy. Whenever fluorescence imaging was performed, photographs were taken using a digital camera (Canon Eos 5D Mark II, Canon Inc., Tokyo, Japan). All flap photographs were taken from the same place and angle. The obtained images were examined, and necrosis calculations were performed using Image J v1.0 software (Oracle Corporation, America). Additionally, in the dorsal flap group, the flow velocities of the flaps were calculated using the SPY-Q (Novadaq Technologies Inc., Canada) during all imaging processes. Finally, all flaps were subjected to histopathological examination.

## 3 Results

### 3.1 The problems encountered in indocyanine green angiography

#### 3.1.1 Inability to evaluate perfusion clearly

**Fig. 2:**
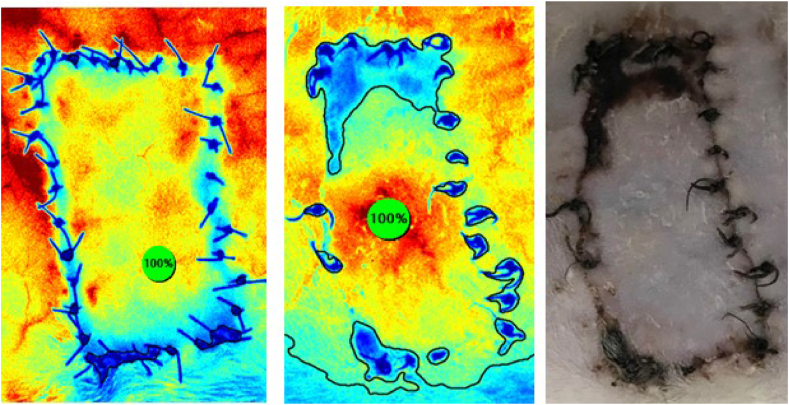
The images illustrate the DIEP flap 0^th^ day ICG angiography image, 3rd day ICG angiography image and 3rd day photograph respectively. The flap appearing to have no filling defects on 0^th^ day due to reflections from the bottom was found to have 19.7 % necrosis on the postoperative 3rd day.

**Fig. 3:**
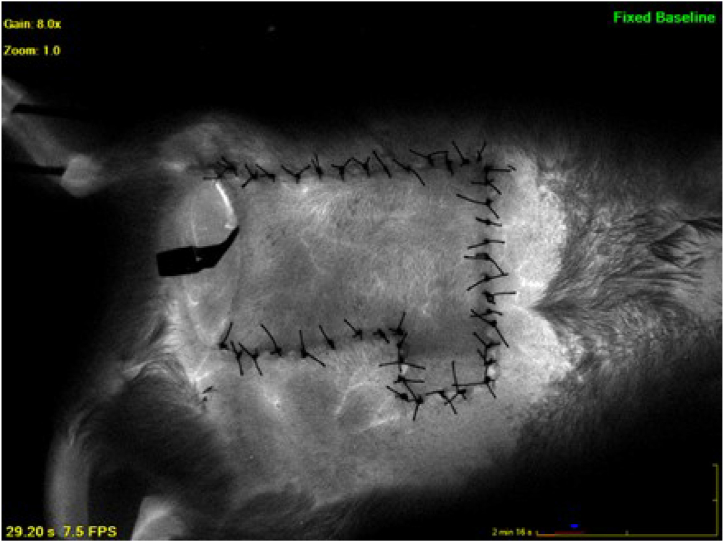
In the elevated epigastric flap, reflections from the bottom of the flap were noticeable, despite the clamping of the pedicle and the use of a silicone background.

The evaluation of perfusion was unclear in 75 % of rats in the first group (n = 3), in all rats in the second group (n = 4), and in 50 % of rats in the third group (n = 4). The DIEP flap, which initially showed no filling defect on the 0^th^ day measurement, was reassessed on the 3rd day using photographs and angiography images, resulting in a calculated necrosis rate of 19.7 % (Fig. 2). In the epigastric flap model, despite clamping the pedicle after flap elevation and using a sterile silicone background, reflections from the interior parts could not be prevented (Fig. 3). Comparatively, the image quality was better in the dorsal flaps than in the abdominal flaps. However, even with the silicone background, reflections could not be completely eliminated, especially in the middle and distal parts (Fig. S2).

#### 3.1.2 Failure to determine the threshold value to predict necrosis rates

While not encountered in first and third group, this problem was observed in 75 % of the rats in the second group (n = 3). Postoperative necrosis was compared, and it was observed that the necrotic regions seen in the photographs corresponded to different threshold values in the SPY images. In a rat, it was observed that determining the perfusion threshold value (*contour level*) in the SPY device as 25 % was sufficient to obtain the necrosis image in the photograph. However, results were variable. For instance, while a perfusion threshold value as low as 13 % was enough to capture the necrosis rate in the photograph for one rat, it had to be increased to 70 % in another rat (Figs. S3–S4).

#### 3.1.3 A part of the flap survived as graft

After suturing the flaps without using any background, certain portions of the flaps were able to survive as grafts. When comparing the postoperative necrosis of the flaps, this issue was observed in two rats from group 1 and in all rats from group 2 who underwent surgery without the use of a background material. However, this problem did not occur in any rats from group 3 due to the implementation of a silicone or aluminum foil background. It was also noted that the areas of the DIEP flap identified as filling defects and necrotic in the postoperative 3rd day angiography did not appear as necrosis when examining the photographs of the same rat (Fig. S5).

### 3.2 Random pattern dorsal flap model using aluminum background

**Fig. 4:**
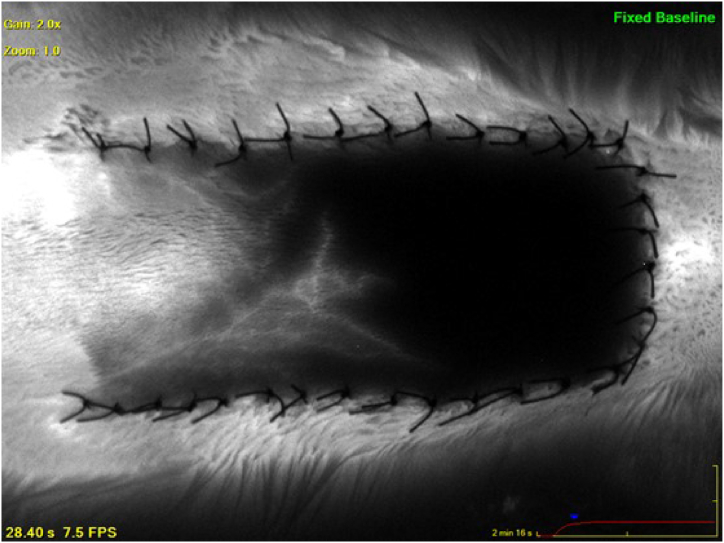
When aluminum foil was used as a background, it was observed that the image was considerably clear, there was no reflection from the interior, and the middle and distal parts were clearly visible.

Sterile aluminum foil was utilized as a background in half of the rats from group 3 (n = 4). The resulting images displayed notable clarity, with no reflections observed from the inner parts. Furthermore, the middle and distal portions were distinctly visible (Fig. 4).

**Fig. 5:**
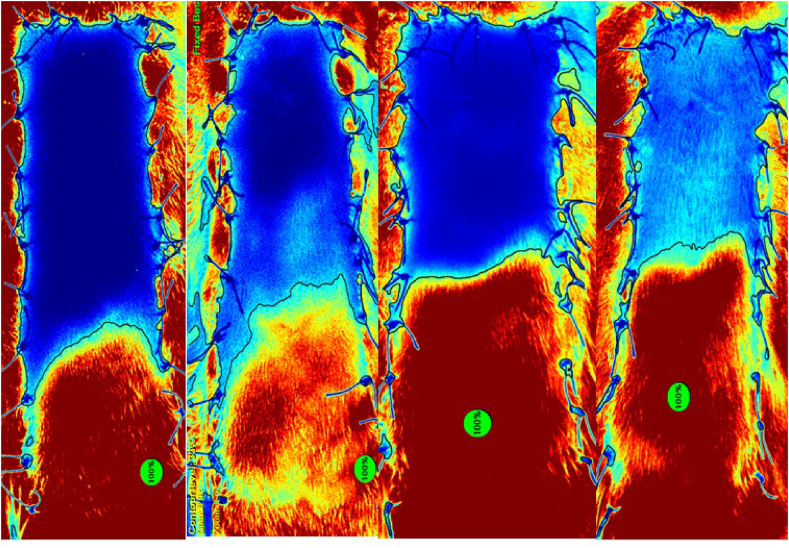
The images of the random pattern dorsal flap with aluminum background of the same rat, performed with the SPY device on the 0^th^ minute, 30th minute, 5th day and 7th day, respectively. It was determined that there was no reflection from the bottom even with repetitive shots. The 5th and 7th day examinations revealed that the aluminum foil remained in its place in and the imaging was considerably clear.

In this group, repetitive imaging was conducted on the postoperative 30th minute, 5th day, and 7th day. It was observed that the images remained undistorted and were not affected by the images captured in the previous shots (Fig. S6, Fig. 5).

**Fig. 6:**
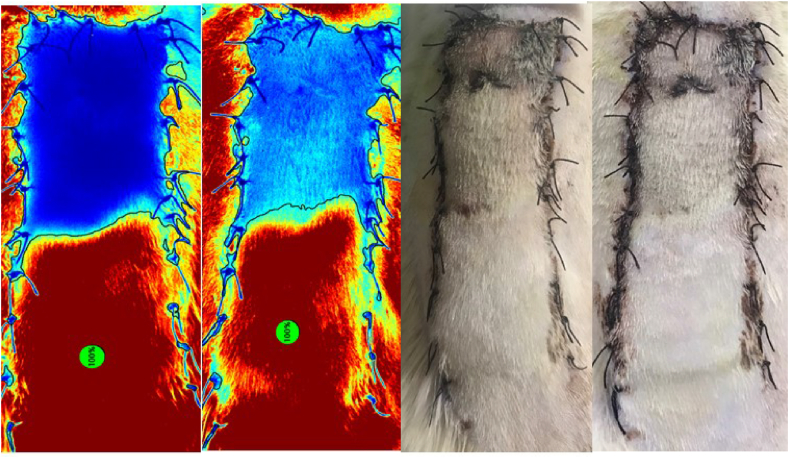
Illustration of images of the same rat; 5th day SPY image, the 7th day SPY image, the 5th day photograph and the 7th day photograph, respectively. It is noteworthy that the 5th day and 7th day images obtained both in indocyanine angiography and digital photography were very similar.

While photographs were compared to the angiographic images, it was observed that the necrosis rates were consistent with each other (Fig. S7). In addition, calculated necrosis rates and angiography images were remarkably similar on the postoperative 5th and 7th days (Fig. 6).

Flow analysis using the SPY-Q software was conducted in four rats within the dorsal flap model, utilizing an aluminum background. The “Region” section in the SPY-Q software was used, the dorsal flaps were selected rectangularly, and the fluorescence intensity (*ingress*) and fluorescence filling rate (*ingress rate*) in the flaps were measured (Fig. S8).

The average ingress values on the postoperative 0^th^, 5th and 7th days were determined to be 2.75, 60.5, and 83 units, respectively. Similarly, the average ingress rates were found to be 0.075, 3.75, and 7.37 units/second on the same respective days. Throughout the experiment, the impact of the aluminum foil used in this model on the rats was observed. Daily monitoring included observations of feeding, mobility, and weight gain. No harmful effects of the aluminum foil were observed. On the postoperative 7th day, the examination conducted after euthanasia revealed that the aluminum foil, despite being damaged, remained in place without any signs of infection or seroma formation (Fig. S9). Furthermore, the histopathological examination did not identify any granulomatous inflammation or foreign-body giant cell formation (Fig. S10).

## 4 Discussion

Despite evolving information on flap anatomy and advanced surgical techniques, ischemia and necrosis are still important problems [1,6]. There are only limited studies in the literature investigating flap perfusion using indocyanine green angiography, which is a useful non-invasive imaging method. *Fourman* et al. stated that ICG green angiography was more successful then laser doppler at predicting necrosis at an early stage in McFarlane flap [6]. *Wang* et al. emphasized that indocyanine green angiography performed with the SPY system was an important method for assessing the flap perfusion [7].

The amount of indocyanine green dose used in rats varies in studies. *Fourman* et al. used ICG at 2.5 mg/mL concentration in a volume of 0.04 mL and *Wang* et al. used ICG at 4.16 mg/mL concentration in a volume of 0.05 mL [6,7]. In our study, we determined that the administration of ICG dye in a volume of 0.1 mL at a concentration of 0.25 mg/mL for each angiography was sufficient. This low dose prevented overlapping of images and deterioration of image quality because of non-metabolized dye remaining from previous images during repetitive shots. Also, the use of a lower amount of dye reduces the possibility of ICG toxicity.

The analysis of perfusion performed by the SPY device enables early detection of tissue perfusion under risk and allows simultaneous intervention [5]. In this software, it can be predicted that the regions below a certain percentage will be necrotic, when the region having the maximum supply in the flap corresponds to 100 %. The study conducted by *Moyer* et al. determined that all regions below 25 % became necrosis, all regions over 45 % were vital, and the viability rates of regions between 25 and 45 % varied in mastectomy flaps [8]. However, these calculations are based on human beings’ studies. One of the findings we encountered in our study was that fluorescent reflections from internal organs made correct perfusion measurements difficult in small animals such as rats. Especially in the DIEP flap model, the reflections from the abdominal organs (especially the liver), did not allow making an accurate analysis (Fig. 2). ICG angiography can detect changes in the blood flow from the skin to a depth of 2 cm [9]. ICG dye is metabolized in the hepatobiliary system [10]. Therefore, reflections of ICG coming from the liver will be enough to interfere with imaging in abdominal flap models. We tried to block the reflection with a background in our study. The image quality increased after using the background in groups 1 and 3. However in the DIEP flap group (group 2), it was not possible to use a background due to the possible mechanical damage to the perforator artery. Also, the threshold value for the prediction of estimated necrosis in abdominal flap model, even with a background material, was found to be quite variable and impractical (wide range 13–70 %) (Figs. S3–S4).

Another important issue is that a part of the flap survived as a graft after its adaptation (Fig. S5). The problem was observed only for the rats without a background. This indicates that the tissues in these areas were not supplied by the random or axial pattern, but by diffusion from the underlying tissues and survived as grafts. When a background material was used, the flap was disconnected from the wound bed, feeding with diffusion from the bottom was prevented, and the shape of necrosis detected by the ICG imaging was the same as that detected in the photographs (Fig. S7, Fig. 6). *Hammond* et al. reported that a portion of the flap was retained as a graft [11]. Many materials such as silicone or polypropylene have been used to disconnect the flap from its bed [6]. *Kelly* et al. designed a tubed flap for the separation of flap from the wound bed to prevent healing as a graft [12]. In a study conducted by *Greenwood* et al. following a partial thickness graft placement, in vivo real-time blood flow was observed first after 4 days [13]. Since revascularization does not start on the postoperative 3rd day yet, ICG angiography on that day exhibits no vascularization pattern in the areas which are survived as graft and these areas are assessed as necrosis. However, as the same areas are supplied by diffusion from below, they will not appear necrotic in the photographs taken. Although the previous studies indicated that part of the elevated flap was survived as a graft, our study supports these findings by demonstrating it with ICG angiography.

In addition to separating the flap from the wound bed to prevent healing as graft, *Fourman* et al. used sterile silicone layers to stabilize the appearance and changes in blood flow [6]. However, in our study, silicone background was observed not to provide sufficient isolation for ICG images (Fig. 3-S2). But, in the dorsal flap model with sterile aluminum foil background, there was no reflection from the bottom and the filling and vascularization pattern of the flap could be clearly observed, even with repetitive shots (Fig. 4, Fig. 5). There are certain reasons why aluminum foil is used as a background in our study. While sterile silicone or polypropylene sheets with a thicker design may apply pressure to the pedicle and the flap due to their thickness, the tension to the pedicle and flap could be maintained lower with using aluminum foil due to its thin structure. Fluorescent rays of the ICG dye with a wavelength of 75–3000 nm are in the near-infrared range [14,15]. Aluminum has the ability to reflect up to 97 % in the near-infrared light region, where silicone has high transmittance [16,17]. The higher reflectance of aluminum instead of transmitting the fluorescence caused by indocyanine green dye gives it a distinct advantage over silicone. Aluminum foil is affordable and easily available, and it can be easily sterilized. In accordance with the reasons mentioned above, we think that aluminum foil could be suggested as an ideal background for certain experimental flap models. It is known that the backgrounds might cause infection, seroma, congestion, and inflammation [6]. Our results on feeding, mobility, and weight gain of the rats and the histopathological evaluation showed that it is safe to use the aluminum foil for at least an observation period of 7 days (Figs. S9–S10).

The measurement of necrosis in experimental flap models is generally conducted by planimetric analysis [1]. The measurement can be performed with regularly taken photographs or by evaluating the appearance of the necrosis from outside [18]. In our study, in the dorsal flaps in which aluminum foil background was used, it was observed that the flap photographs and their images obtained by the SPY device overlapped with each other (Fig. S7). Suture reactions, damage to the flap caused by the surgical instruments during the operation, and hair regrowth of the necrotic areas of the rat in a very short time reduce the sensitivity of the measurements. We think that, examining the image obtained by the optimal non-invasive imaging method instead of taken photographs would increase the sensitivity in the measurement of the precise necrosis rates in the postoperative period. In addition, based on the fact that the necrosis rates and the images obtained were very similar to each other on the postoperative 5th and 7th days, it could be suggested that five days would be sufficient for the evaluation of necrosis in dorsal flap models. In our previously published study, we utilized angiography images in the DFM and observed that perioperative taurine administration resulted in significantly higher values of Ingress and Ingress rate, indicating a gradual enhancement in flap perfusion and perfusion rate as assessed by the SPY-Q software in an experimental rat flap model. Furthermore, the findings demonstrated that perioperative taurine treatment effectively decreased necrosis rates while increasing fluorescence density, fluorescence filling rate, and flap filling rates in the DFM [19].

The intensity and velocity of fluorescent entering the flap could be measured using SPY-Q software. While “*the Ingress*” refers to the amount of fluorescent in the tissue, “*the Ingress Rate*” corresponds to the rate at which this amount increases with respect to time, in other words, velocity [20,21]. In our study, flow analyzes in four dorsal flaps using aluminum foil background showed that the average *Ingress* and average *Ingress Rate* values increased as the postoperative day increased. This occurrence could be interpreted as the flap perfusion and the perfusion rate increased with days progressing. As the SPY-Q software allows the analysis for only rectangularly selected areas and does not consider the reflections that came from under the flap with the background use; the rectangularly planned dorsal flap model with the aluminum foil background seems to be ideal for SPY-Q analyzes.

To address the limitations of our study, the less frequent imaging sessions may have limited the observation of flap changes. Imaging was performed 30 min after flap adaptation and on days 3, 5, and 7, in accordance with a careful anesthetic approach that prioritized ethical treatment and minimized the risk of animal loss. The selection of indocyanine green (ICG) imaging times was supported by preliminary experiments and contributed to improved assessment efficiency.

Additionally, the sample size in our study can be considered a limitation. However, the aim of our study is to address the challenges in ICG angiography, which is descriptive in nature. We evaluated indocyanine green angiography in different flap models, and the encountered issues were very clear as either present or not.

## 5 Conclusion

Achieving optimal ICG angiography requires precise prediction and interpretation of results. Our study suggests that adjustments to ICG imaging of the flap are important for developing a standardized approach to investigating flap perfusion and viability while reducing the influence of external factors on the results. Utilizing a background in this modified technique is essential to minimize reflections from internal structures. We found that using an aluminum foil background is both effective and safe for experimental animals. Nevertheless, further research comparing different background materials could provide more comprehensive insights into their relative advantages and limitations. Building on the insights from our study, these advancements will enhance and simplify flap imaging in experimental models, leading to improved understanding and precision in assessing flap viability. These developments not only support the translation of findings into clinical practice but also contribute valuable information on the pathophysiological mechanisms influencing flap viability.

## Ethics statement

This study was reviewed and approved by Ankara University Animal Experiments Local Ethics Committee, with the approval date: July 22, 2020 and number: 2020-13-124.

## Funding

This research did not receive any specific grant from funding agencies in the public, commercial, or not-for-profit sectors.

## Data availability statement

Data will be made available on request.

## CRediT authorship contribution statement

**Mert Ersan:** Writing – review & editing, Writing – original draft, Visualization, Resources, Methodology, Investigation, Formal analysis, Data curation, Conceptualization. **Burak Kaya:** Writing – original draft, Visualization, Validation, Resources, Methodology, Investigation, Data curation, Conceptualization. **Arda Özdemir:** Writing – original draft, Visualization, Validation, Software, Resources, Methodology, Investigation, Data curation. **Aygül Durdurur Çin:** Writing – review & editing, Visualization, Validation, Software, Methodology, Investigation, Formal analysis, Conceptualization. **Hakan Ergün:** Writing – review & editing, Writing – original draft, Visualization, Validation, Supervision, Resources, Project administration, Methodology, Investigation, Formal analysis, Data curation, Conceptualization.

## Declaration of generative AI and AI-assisted technologies in the writing process

During the preparation of this work the author(s) used ChatGPT – OpenAI in order to improve readability and language. After using this tool/service, the author(s) reviewed and edited the content as needed and take(s) full responsibility for the content of the publication.

## Declaration of competing interest

The authors declare that they have no known competing financial interests or personal relationships that could have appeared to influence the work reported in this paper.
