# Supplementary material for: Challenges in flap monitoring with indocyanine green angiography in experimental models
Source: Heliyon. 2024 Aug 14;10(16):e36034. doi: 10.1016/j.heliyon.2024.e36034 (PMC11388392; doi:10.1016/j.heliyon.2024.e36034)
Supplement: Multimedia component 1 [file mmc1.docx]

1. **SUPPLEMENTARY DATA**

Figure S1: Preparation of DIEP flap (left) and abdominal perforators in rats (right).

Figure S2: In elevated dorsal flaps, the image was better than abdominal flaps. However, despite the use of a silicon background, reflections from the bottom could not be completely prevented. Reflections can be seen especially in the middle and distal parts.

Figure S3: It was observed that the perfusion threshold value (contour level) should be adjusted to 13% to obtain the image on the 3rd day photograph of that rat.

Figure S4: It was seen that the perfusion threshold value (contour level) should be adjusted to 70% to obtain the image on the 3rd day photograph of a different rat.

Figure S5: The regions that appeared as necrosis in SPY angiography on the 3rd day- even at a low threshold value of 16%-, were observed to be vital in the photograph.

Figure S6: Image of the same rat was taken on day 0, 30 minutes, 5th day and 7th day. The picture of 7th day shot displayed that image was considerably clear and did not interfere with the images of previous shots.

Figure S7: When the 7th day indocyanine angiography image was compared to the 7th day photograph for the same rat, it was seen that the images were consistent with each other.


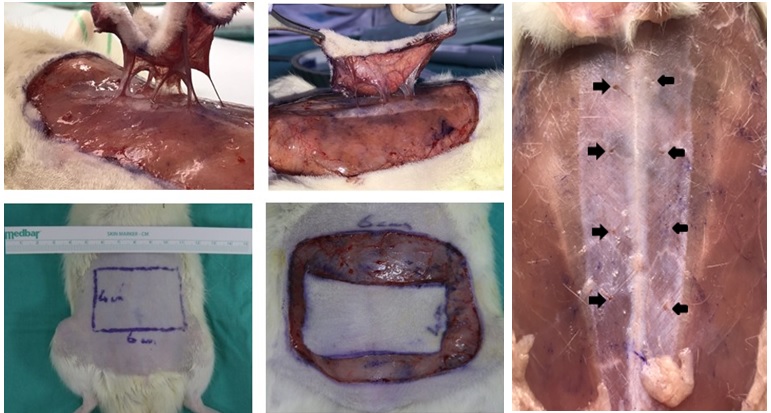


Figure S1: Preparation of DIEP flap (left) and abdominal perforators in rats (right).


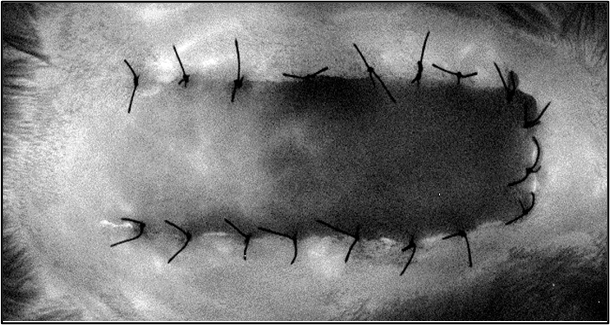


Figure S2: In elevated dorsal flaps, the image was better than abdominal flaps. However, despite the use of a silicon background, reflections from the bottom could not be completely prevented. Reflections can be seen especially in the middle and distal parts.


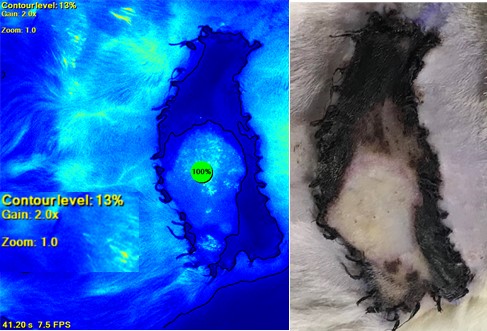


Figure S3: It was observed that the perfusion threshold value (contour level) should be adjusted to 13% to obtain the image on the 3^rd^ day photograph of that rat.


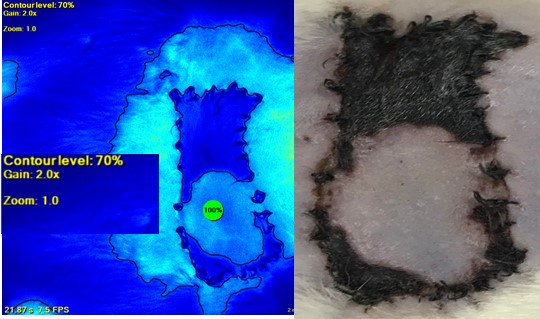


Figure S4: It was seen that the perfusion threshold value (contour level) should be adjusted to 70% to obtain the image on the 3^rd^ day photograph of a different rat.


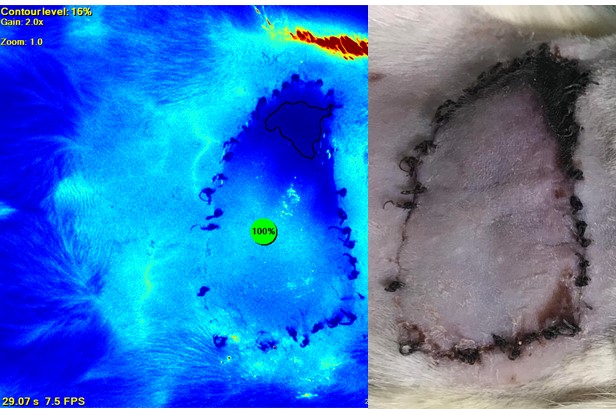


Figure S5: The regions that appeared as necrosis in SPY angiography on the 3^rd^ day- even at a low threshold value of 16%-, were observed to be vital in the photograph.


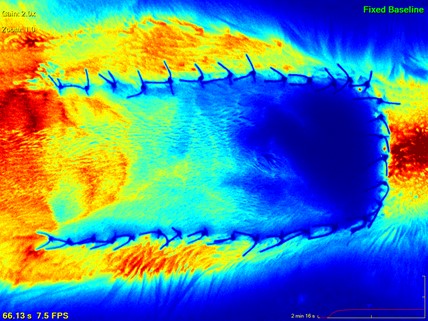


Figure S6: Image of the same rat was taken on day 0, 30 minutes, 5^th^ day and 7^th^ day. The picture of 7^th^ day shot displayed that image was considerably clear and did not interfere with the images of previous shots.


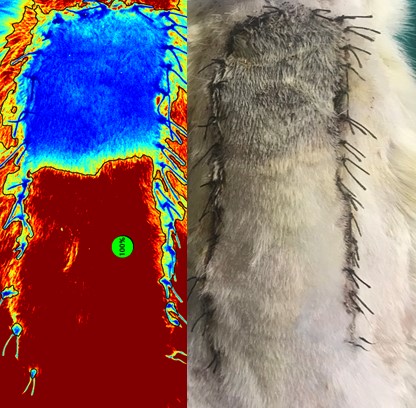


Figure S7: When the 7^th^ day indocyanine angiography image was compared to the 7^th^ day photograph for the same rat, it was seen that the images were consistent with each other.


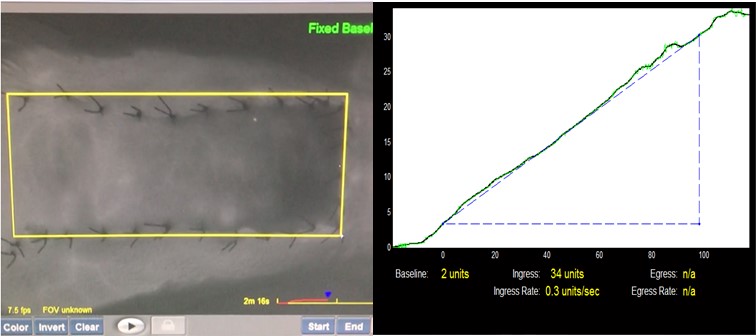


**(a)**

**(b)**

Figure S8: Fluorescence intensity (Ingress) and fluorescence filling rate (Ingress Rate) in the selected tissue can be calculated by rectangularly selecting the desired region using the "Region" tab of the SPY-Q program.


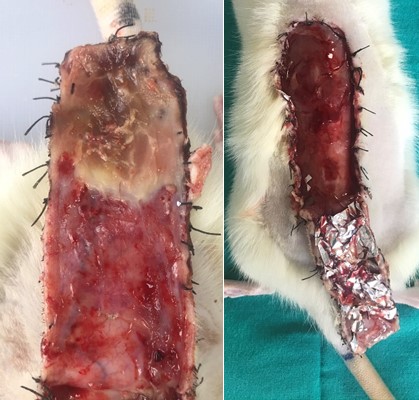


Figure S9: On the postoperative 7^th^ day, the examination performed after euthanasia demonstrated that the aluminum foil despite being damaged, remained in its place without any indication of infection or seroma.


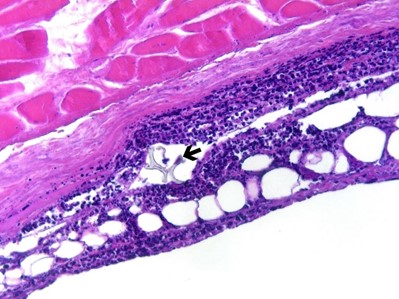


Figure S10: The picture displays a gray colored foreign–body (aluminum foil) between the neutrophil leukocytes (marked by an arrow in the picture with magnification: x100, stain: HE). A granulomatous response or foreign–body giant cells were not found in those sections.
